# Normal Hearing Sensitivity at Low-to-Middle Frequencies with 34% Prestin-Charge Density

**DOI:** 10.1371/journal.pone.0045453

**Published:** 2012-09-21

**Authors:** Tetsuji Yamashita, Jie Fang, Jiangang Gao, Yiling Yu, Marcia Mellado Lagarde, Jian Zuo

**Affiliations:** Department of Developmental Neurobiology, St. Jude Children's Research Hospital, Memphis, Tennessee, United States of America; University of Southern California, United States of America

## Abstract

The mammalian outer hair cells (OHCs) provide a positive mechanical feedback to enhance the cochlea's hearing sensitivity and frequency selectivity. Although the OHC-specific, somatic motor protein prestin is required for cochlear amplification, it remains unclear whether prestin can provide sufficient cycle-by-cycle feedback. In cochlear mechanical modeling, varying amounts of OHC motor activity should provide varying degrees of feedback efficiency to adjust the gain of cochlear amplifier at resonant frequencies. Here we created and characterized two new prestin-hypomorphic mouse models with reduced levels of wild-type prestin. OHCs from these mice exhibited length, total elementary charge movement (*Q*_max_), charge density, and electromotility intermediate between those of wild-type and prestin-null mice. Remarkably, measurements of auditory brainstem responses and distortion product otoacoustic emissions from these mice displayed wild-type like hearing sensitivities at 4–22 kHz. These results indicate that as low as 26.7% *Q*_max_, 34.0% charge density and 44.0% electromotility in OHCs were sufficient for wild-type-like hearing sensitivity in mice at 4–22 kHz, and that these *in vitro* parameters of OHCs did not correlate linearly with the feedback efficiency for *in vivo* gain of the cochlear amplifier. Our results thus provide valuable data for modeling cochlear mechanics and will stimulate further mechanistic analysis of the cochlear amplifier.

## Introduction

In mammals, outer hair cells (OHCs) provide a nonlinear, positive mechanical feedback in the cochlea to increase hearing sensitivity and frequency selectivity. Both somatic and hair bundle motilities have been proposed to explain the mechanical amplification provided by the cochlea [1]. According to the somatic motor hypothesis, the somatic motor protein prestin undergoes voltage-dependent conformational changes, which are manifested as non-linear capacitance (NLC) that produces changes in the length of OHCs (i.e., electromotility) to enhance sound-induced vibrations of the organ of Corti.

Targeted deletion of prestin in mice (*prestin−/−* mice) causes loss of electromotility and a 40 to 60 decibel (dB) loss of hearing sensitivity [2], [3], consistent with the contribution of the cochlear amplifier in mammals. However, the absence of prestin also produces shorter and less stiff OHCs that lead to considerable differences in passive cochlear mechanics relative to wild-type mice and make it difficult to assess the role of prestin as the amplifier [2], [4]. In support of prestin's role in cochlear amplification, the introduction of the V499G/Y501H (499) mutation into the *prestin* locus, which almost completely abolishes OHC electromotility (∼7.5% of that in wild-type controls) without producing changes in OHC length and stiffness, also causes a 40 to 60 dB loss of hearing sensitivity [5]. In addition, electrically evoked basilar membrane (BM) displacements in *Tecta^ΔENT/ΔENT^* cochlea, in which hair bundle contribution to amplification is disabled near threshold, are as sensitive and tuned as those acoustically and electrically elicited in wild-type controls [6]. Together these studies provide strong evidence that prestin-based OHC electromotility is the basis for cochlear amplification.

However, as in all feedback systems, the gain at the resonant frequency must be adjusted in the cochlear amplifier. In Patuzzi's model [7], if prestin is the OHC motor, it may accommodate to the need for varying degrees of mechanical feedback to allow proper function of the amplifier. That is, if the quantity of functional prestin is proportional to the degree of feedback efficiency, the gain of the amplifier should decrease, from ∼45 to 0 dB, nonlinearly with decreasing prestin motor activity, from 100% to 0%. The *prestin* heterozygous knockout (*prestin+/−*) mouse was previously used to study this relationship; however, it has compensatory upregulation of prestin expression and thereby wild-type–like hearing [8]. Therefore, mice with intermediate levels of prestin activities are needed to reveal the full dynamic range of the feedback system. In addition, analysis of intermediate levels of prestin can reveal the critical amount of prestin activities required for proper amplification *in vivo*.

Here, we created novel knock-in mice expressing intermediate levels of wild-type prestin and showing intermediate levels of total elementary charge movement (*Q*_max_), charge density and electromotility in isolated OHCs. The hearing sensitivity and cochlear function were analyzed in these mutant mice to correlate with nonlinear capacitance (NLC), charge density, and electromotility in isolated OHCs. Our findings shed light on the mechanisms of cochlear amplification and provide *in vivo* data for modeling the cochlear amplifier.

## Materials and Methods

### Animals

Mice were housed under a 12 h light/dark cycle with free access to food and water. All surgical procedures were performed under anesthesia with an intraperitoneal (IP) injection of Avertin (0.5 mg/kg body weight). All of the protocols performed in this study were approved by the Animal Care and Use Committee of St. Jude Children's Research Hospital.

### Gene targeting

**Figure 1:**
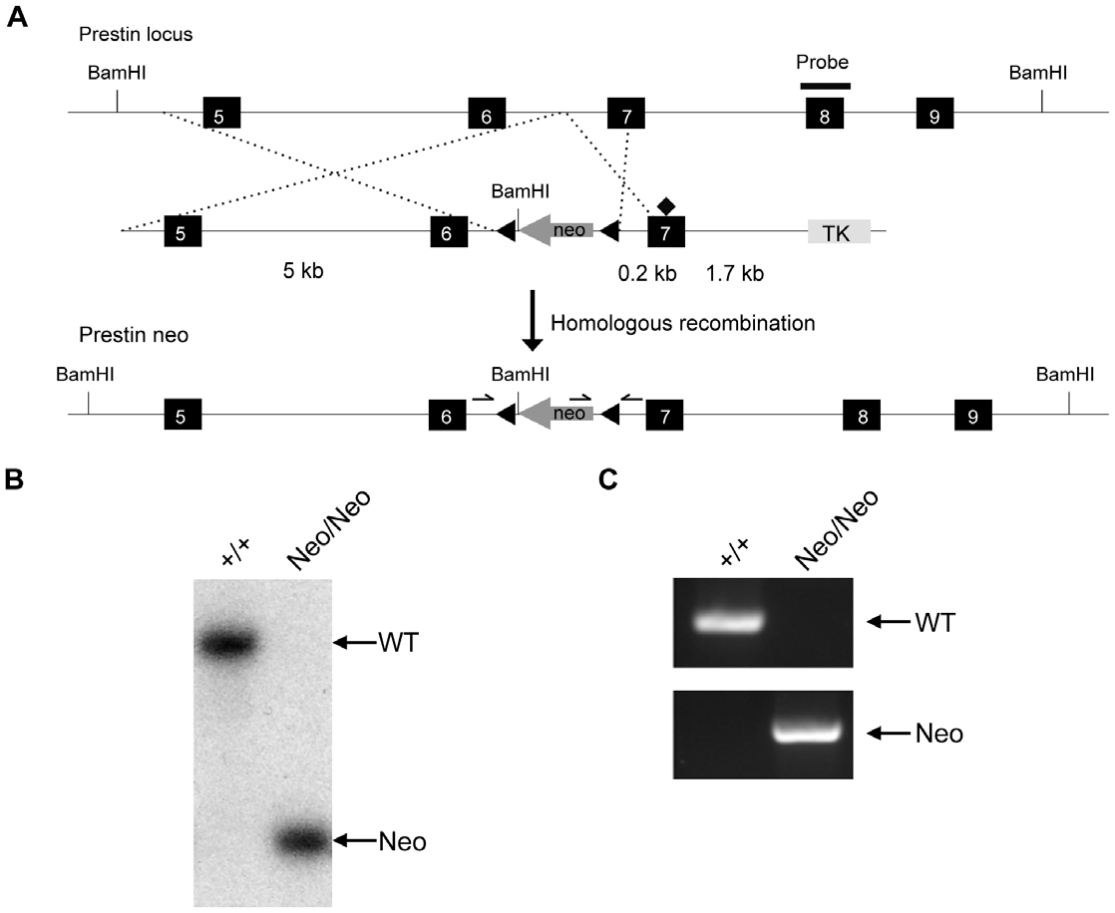
*Prestin* knock-in mice. (***A***) Targeted Neo *prestin* knock-in allele. Solid rectangles represent exons 5 through 9. Diamond indicates the C1 mutation [9]. A neo-selectable marker was inserted into intron 6 of the *prestin* gene in ES cells by homologous recombination. (***B***) Genomic Southern blot analysis of *Neo/Neo* mice. Genomic DNAs from *+/+* and *Neo/Neo* tails were digested with *Bam*HI and a specific probe indicated in (***A***) was used to detect a 12-kb band in the *+/+* allele and an 8-kb band in the targeted allele. (***C***) PCR-based genotyping of *+/+* and *Neo/Neo* mice using 3 primers is indicated in (***A***). Wild-type mice showed a 242-bp band; the *Neo/Neo* mice showed a 400-bp band.

Prestin knock-in mice were generated as previously described [9]. Briefly, the targeting vector containing K233Q/K235Q/R236Q (C1) mutation was constructed in PL452 (Fig. 1*A*) and transfected into 129/SvEvTac (129S6) ES cells (TL-1) as described previously [9]. Sequencing analysis by using the homologous recombinant ES cells revealed that 2 of 6 homologous recombinant ES cell clones showed no C1 mutations, consistent with the notion that the homologous recombination event occurred between the neomycin phosphotransferase (neo) cassette and the C1 mutation (Fig. 1*A*; data not shown). To create chimeric mice, the ES cells obtained were injected into the C57BL/6 blastocysts and embryos were transplanted into foster mice.

### Genotyping

The following polymerase chain reaction (PCR) primers were designed to detect the neo cassette in intron 6 (inserted as a selectable marker for ES cell screening): wild-type upstream primer, 5′- GGC AGA GTC AGC AAA ACT CC -3′; knock-in upstream primer, 5′- CCG GTG GAT GTG GAA TGT GTG CGA GGC CAG AG -3′; common downstream primer, 5′- AAC CTT GGC CAT GCT TAC AC - 3′, yielding PCR products of 400 bps for the neo knockin allele and 242 bps for the wild-type allele. The *prestin*−/− allele was genotypically confirmed as the deletion of exons 3 to 6 containing prestin initiation codon, as described previously [2].

### Histologic analysis

Inner ears were fixed overnight in 4% paraformaldehyde in 0.1 M phosphate buffer (pH 7.4) and then decalcified in 10% EDTA (pH 7.4) for 24–48 h at 4°C. Cochleae were carefully dissected from the decalcified inner ear.

Cochlear location was recorded by using a frequency map described by Mueller et al [10] and modified by Taberner et al [11]. The modified equation used was *f* (kHz) = 9.8×(10*^d^* ^×0.92^−0.68), where *d* is the normalized distance from the cochlear apex (0–1) [12].

### Immunostaining

Primary antibodies used were goat anti-prestin antibody (N-20; 1∶200 dilution, Santa Cruz Biotechnology, Santa Cruz, CA), rabbit anti-myo6 antibody conjugated to Alexa647 (1∶100 dilution, Proteus Bioscience), and Alexa Fluor 546 phalloidin (1∶100 dilution, Invitrogen, Carlsbad, CA). Immunofluorescence was visualized by adding Alexa Fluor 488 chicken anti-goat IgG (H+L) (Molecular Probes, Eugene, OR). Counter-staining of nuclei was performed using 4′,6-diamidino-2-phenylindole (DAPI, SIGMA, St-Louis, MO).

### Measurement of OHC length in cochlear whole mounts

To measure OHC length, whole mounts of cochleae were prepared for each genotype; a frequency map was generated; and prestin, Myo6, nuclei, and F-actin were stained as described above. Fluorescence images were analyzed with a Zeiss Axiophot2 microscope equipped with a 40× oil immersion and 1.4 NA objective and were captured at 0.6 µm intervals from the upper to lower edges by using a LSM510 Meta confocal laser scanning image system (Carl Zeiss, Jena). Optical sections were obtained at depth intervals of 0.6 µm. After a 3D reconstruction of cochlear whole mounts, the distance from the cuticular plate to the center of the nucleus was measured as OHC length drawing a line along the center of the cylindrical OHCs, using the Imaris 6. 1. 5. software (Bitplane, Zurich, Switzerland).

### Measurement of NLC

Cochleae were harvested from mice of different genotypes at postnatal days 21–24 (P21–P24). The organ of Corti was dissociated from the apical region of the cochlea by digestion with 1 mg/mL dispase (Worthington Biochemical, Lakewood, NJ) for approximately 10 min. OHCs were isolated by triturating gently twice with a fire-polished plastic pipette. Isolated OHCs were placed in a chamber mounted on an inverted microscope with an external bath block. Healthy, cylindrical OHCs were selected to measure nonlinear membrane capacitance. OHCs were occasionally swollen in the course of experiments and the data points were excluded.

Patch pipettes were created by using a pipette puller (P2000, Sutter Instruments, Novato, CA). OHCs in the approximately 4 to 16 kHz region were used for measurements (data not shown). The resistance of the pipettes used was 2 to 3 MΩ when filled with an intracellular medium. NLC of cells was recorded by whole-cell voltage patch clamp at room temperature, using an Axon patch 200B instrument (Union City, CA). Internal and external bath block solutions were prepared as previously described [13]. The internal solution was 140 mM CsCl, 2 mM CaCl_2_, 5 mM EGTA, and 10 mM K-HEPES. The external solution was 140 mM NaCl, 5 mM CsCl, 2 mM MgCl_2_, 1 mM CaCl_2_, 2 mM CoCl_2_, and 10 mM Na-HEPES. The osmolarity was adjusted to 300 mOsm/kg by adding glucose. The pH was adjusted to 7.4. NLC was measured by using jClamp (SciSoft, CT). Patched cells were measured by using a continuous high-resolution 2-sine stimulus wave technique [14], [15] with a voltage ramp from −150 to +120 mV after whole-cell configuration was established. The nonlinear capacitance C_m_(V) data were fitted by using a derivative of the two-state Boltzmann function that involves linear capacitance *C*_lin_, maximum nonlinear charge transfer *Q*_max_, the voltage at half-maximum charge transfer *V*_1/2_, and slope factor α (Eq. 1). Data analysis and curve fitting used the software Igor Pro 6.0 (WaveMetrics, Lake Owego, OR).
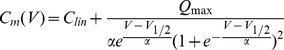
(1)Data for each genotype were collected from at least 3 independent mice.

### OHC electromotility

Healthy, cylindrical OHCs from the 4–16 kHz region were selected to measure changes in their length. To evoke OHC electromotility, OHCs were held at −70 mV and voltage changes from −150 mV to 120 mV were applied in 30 mV increments under the whole-cell voltage–clamp mode. OHCs before and during the experiments were observed by using Zeiss Axio Imager A1 inverted microscope (Carl Zeiss, Jena, Germany) equipped with 63× water immersion/1.2 numerical aperture (NA) objective and a DAGE-MTI CCD-100 video camera (DAGE-MTI, Michigan City, IN). The resolution of images was 720×480 pixel per inch and frame rate was 29.97 frames/s. Movements of the cuticular plate's position were traced by using Image J (http://rsbweb.nih.gov/ij/, [16]). The maximum amplitude of the traced wave was defined as an absolute value of amplitude of electromotility. The distance between the cuticular plate and the point at which the patch pipette was attached was measured along the lateral wall of the OHC as the OHC length at holding potential.

### Measurement of total surface area of lateral membrane of isolated OHCs

OHCs were confirmed to be positioned parallel to the bottom of culture dishes under phase contrast and axial lengths of the OHC lateral walls were measured as previously described [17]. The OHC diameter was calculated by averaging the widths of cells' midpoints and the widths at the center of nucleus. The surface area of the lateral membrane containing prestin was calculated by *A*_lat_ = π*DL*, where *D* is the diameter and *L* is the length of the membrane containing prestin, because this membrane has the shape of an elongated cylinder in OHCs [17].

### Distortion products otoacoustic emission (DPOAE)

Mice were anesthetized by intraperitoneal injection with Avertin (0.5 mg/kg body weight) and placed on an electric heating pad to maintain body temperature, using a homeothermic blanket system (Harvard Apparatus Ltd). Mice that died or showed signs of middle-ear dysfunction during the course of experiments were excluded from analysis. All recordings were conducted in a sound booth (Industrial Acoustic Company).

For acoustic stimulation and measurements two speakers (*f*_1_ and *f*_2_; EC1) and a microphone (ER-10B, Etymotic Research, Elk Grove Village, IL) were connected to a short flexible coupler tube, and tapered plastic tip that was inserted into the external auditory meatus of mice. The microphone was calibrated in situ with the coupler in measuring position.

Frequency responses of the measurement microphone (ER10B+) at frequencies higher than 22 kHz are lower than those of a reference microphone (ACO-7017; ACO Pacific, Inc., Belmont, CA). Therefore, DPOAE 2*f_1_*−*f_2_* responses were recorded at a frequency of *f_1_* range of 5454–18180 Hz, using the TDT BioSig III system (TDT). Signal duration was 83.88 ms, with a repetition rate of 11.92/s. The *f*_1_ and *f*_2_ responses were passed separately through an RX6 MultiFunction Processor (TDT) for digital/analog conversion to PA5 programmable attenuators. Stimulus intensity was reduced from 70 to 0 dB in 5 dB steps to establish thresholds and was digitally sampled at 200 kHz and averaged from 100 discrete spectra. The signals were delivered through ED1 speaker drivers that fed into the EC1 electrostatic speakers coupled to the ear canal. The resulting ear canal sound pressure was recorded with an ER10B+ low noise microphone (gain 0×) and probe (Etymotic) housed in the same coupler as the *f*_1_ and *f*_2_ speakers. The output of the ER10B+ amplifier was routed directly to an RX6 MultiFunction Processor (TDT) for analog/digital conversion for sampling at 200 kHz. Fast-Fourier transforms (FFT) of averaged responses were generated by using TDT BioSigRP software on the resultant waveform (TDT). Noise floors were determined by averaging the sound levels of 10 frequency bins above and below the 2*f_1_*−*f_2_* frequency bin. No instrumental distortion products were observed in an evaluation of ears postmortem.

### Auditory brainstem response (ABR) testing

ABR waveforms were recorded in a sound booth (Industrial Acoustic Company, Bronx, NY) using subdermal needles positioned in the skull, below the pinna and at the base of the tail and the responses were fed into low-impedance Medusa Digital Biological Amplifier System (RA4L, TDT; 20 dB gain). At each frequency, the stimulus intensity was reduced from 75 to 0 dB in 5 dB steps to determine the threshold dB SPL when the electrical response was just above the noise floor. ABR waveforms were averaged in response to 500 tone bursts. The recorded signals were filtered by a band-pass filter from 300 Hz to 3 kHz.

### Closed field ABR testing

Closed field ABR was measured as described previously [3], with the following modifications. Briefly, calibrated tone bursts were produced with a Tucker-Davis Technologies (TDT) BioSig III system, using a digital/analog converter (TDT, Alachua, FL, RP2.1, 100 kHz sampling rate) at a rate of 21/s at frequencies of 4, 6, 12, 16, 22, 32, and 44 kHz. The generated tone bursts were attenuated with a programmable attenuator (PA5, TDT) and went to ED1 speaker drivers that fed into the EC1 electrostatic speakers (TDT, Alachua, FL). At each frequency, the stimulus intensity was reduced from 70 to 0 dB in 5 dB increments to establish thresholds until no ABR waveforms were observed. ABR waveforms were averaged in response to 500 tone bursts. The recorded signals were filtered by a band-pass filter from 300 Hz to 3 kHz.

### Opened field ABR testing

Calibrated tone bursts were produced using a BioSigRZ system (Tucker-Davis Technologies (TDT), Alachua, FL, RZ6, 200 kHz sampling rate) and delivered through a Multi-Field (MF1) magnetic speaker (TDT) at a rate of 21/s at frequencies of 4, 6, 12, 16, 22, 32 and 44 kHz in a free field configuration. The stimulus sound pressure was calibrated using PCB 377C10 microphone (PCB Piezotronics, Inc. New York, NY). All animals tested were placed 5 cm away from the MF1 magnetic speaker.

### Statistical analysis

GraphPad Prism (Graphpad Software, San Diego, CA) was used for all analyses.

## Results

Insertion of a neo cassette into an intron by gene targeting can alter gene expression by virtue of cryptic splice sites in the cassette, leading to hypomorphic or null alleles [18]. When K233Q/K235Q/R236Q (C1) mutations were introduced into the *prestin* locus, the targeting construct possessed a floxed Neo selection marker in intron 6. 2 of 6 homologous recombinant ES cell clones showed no C1 mutations (data not shown) but had the Neo cassette inserted into intron 6 in an orientation opposite to that of the *prestin* locus (Fig. 1*A*; [9]), as confirmed by genomic Southern and PCR analysis (Fig. 1*B, C*). When these prestin Neo mice were crossed with *prestin−/−* mice [2], both homozygous prestin Neo knock-in mice (*Neo/Neo*) and mice with a single copy of Neo and a prestin knock-out allele (*Neo/-*) were viable and displayed no behavioral abnormalities.

**Figure 2:**
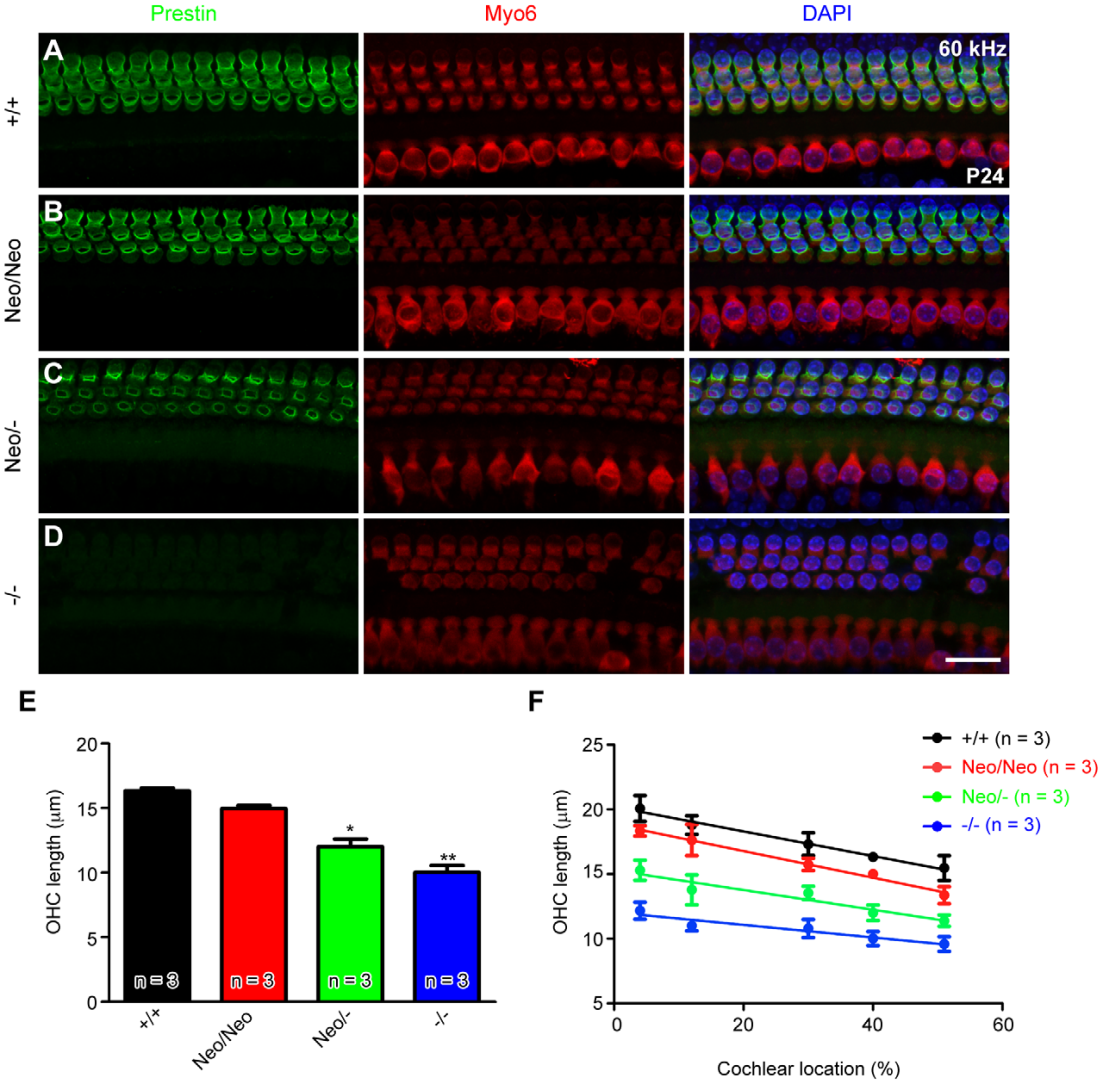
Morphological and immunohistochemical analysis of mutant mice. (***A*** **–** ***D***) Representative immunofluorescence staining with prestin (green) and myosin 6 (red) in whole-mount preparations of basal cochlear turns (corresponding to the 60 kHz region of the +/+ cochlea) in the indicated mouse genotypes at P24. 4′, 6-diamidino-2-phenylindole (DAPI) counterstain is shown in blue. Scale bar: 20 µm. *Neo/Neo* and *Neo/-* mice show no OHC loss or abnormal distribution of prestin. (***E***) Length of OHCs of each genotype at a given location corresponding to 16 kHz region of the +/+ cochlea. Values are the mean ± SEM; **: *P*<0.01, *: *P*<0.05. (***F***) Length of OHCs of each genotype at different locations of the cochlea. The shorter OHCs could reduce the mass of the organ of Corti and result in a higher frequency response for a given location, assuming all other material properties remain the same. The x-axis displays a normalized distance from apex (0%) to base (100%). That is, the locations responding to 4, 6, 12, 16, and 22 kHz in wild-type cochleae correspond to 4, 12, 30, 40, and 51% in a normalized distance from the cochlear apex, respectively. Calculated intercepts for each genotype differed significantly by one-way ANOVA, followed by Student's t test with a Bonferroni correction.

We first investigated whether there was OHC loss in the prestin *Neo/Neo* and *Neo/-* mice. Myo6 was labeled to visualize OHCs and inner hair cells (IHCs) in whole-mount preparations of the basal turns (corresponding to ∼60 kHz region of the *+/+* cochlea) of cochleae at P24. No OHC loss was observed in wild-type (*+/+*), *Neo/Neo*, or *Neo/-* mice, whereas sporadic OHC loss was observed in *prestin−/−* mice, consistent with previous reports [2], [3], [19]. Immunohistochemical analysis showed that there was no difference in the distribution of prestin in *Neo/Neo and Neo/-* mice vs. *+/+* control mice, whereas prestin was not observed in *prestin−/−* mice (Fig. 2*A–D*).

Because the length of OHCs in *prestin−/−* mice has been reported to be only approximately 60% of that in *+/+* mice in the 16 kHz region [2], [20], [21], we examined OHC length in *Neo/Neo* and *Neo/-* mice. OHC length was significantly reduced in P21–24 *prestin−/−* mice (61.4%±3.3% [mean ± SEM]; Fig. 2*E*) compared to +/+ controls in a location corresponding to the 16 kHz region of the *+/+* cochlea, consistent with previous results [2], [20], [21]. OHC length was also significantly reduced in *Neo/-* mice (normalized to +/+, 91.9%±1.6% for *Neo/Neo*, 73.5%±3.7% for *Neo/-*), and there was a linear change in OHC length for different prestin genotypes (*r* ^2^ = 0.91, *P*<0.05; Fig. 2*E*). OHC length decreased from apex to base in a linear manner for the 4 genotypes (*r* ^2^ = 0.65, *P*<0.001 for *+/+* control; *r* ^2^ = 0.77, *P*<0.001 for *Neo/Neo*; *r* ^2^ = 0.58, *P*<0.01 for *Neo/-*; r^2^ = 0.47, *P*<0.01, *−/−*; Fig. 2*F*). Slopes of change in OHC length along the cochlea did not differ among genotypes (analysis of covariance; slopes, *P*>0.05). These data suggest that the gradient of cell length along the cochleae is prestin-independent or that changes of prestin in each mutant affect OHCs uniformly along the cochlear length. No differences were observed in cochlear length among genotypes (6242.0±239.7/µm for +/+, 6209.0±79.8 µm for *Neo/Neo*, 6487.0±90.7 µm for *Neo/-*, and 6855.0±116.2 µm for *prestin*−/−; n = 5 each genotype, P>0.05, ANOVA).

**Figure 3:**
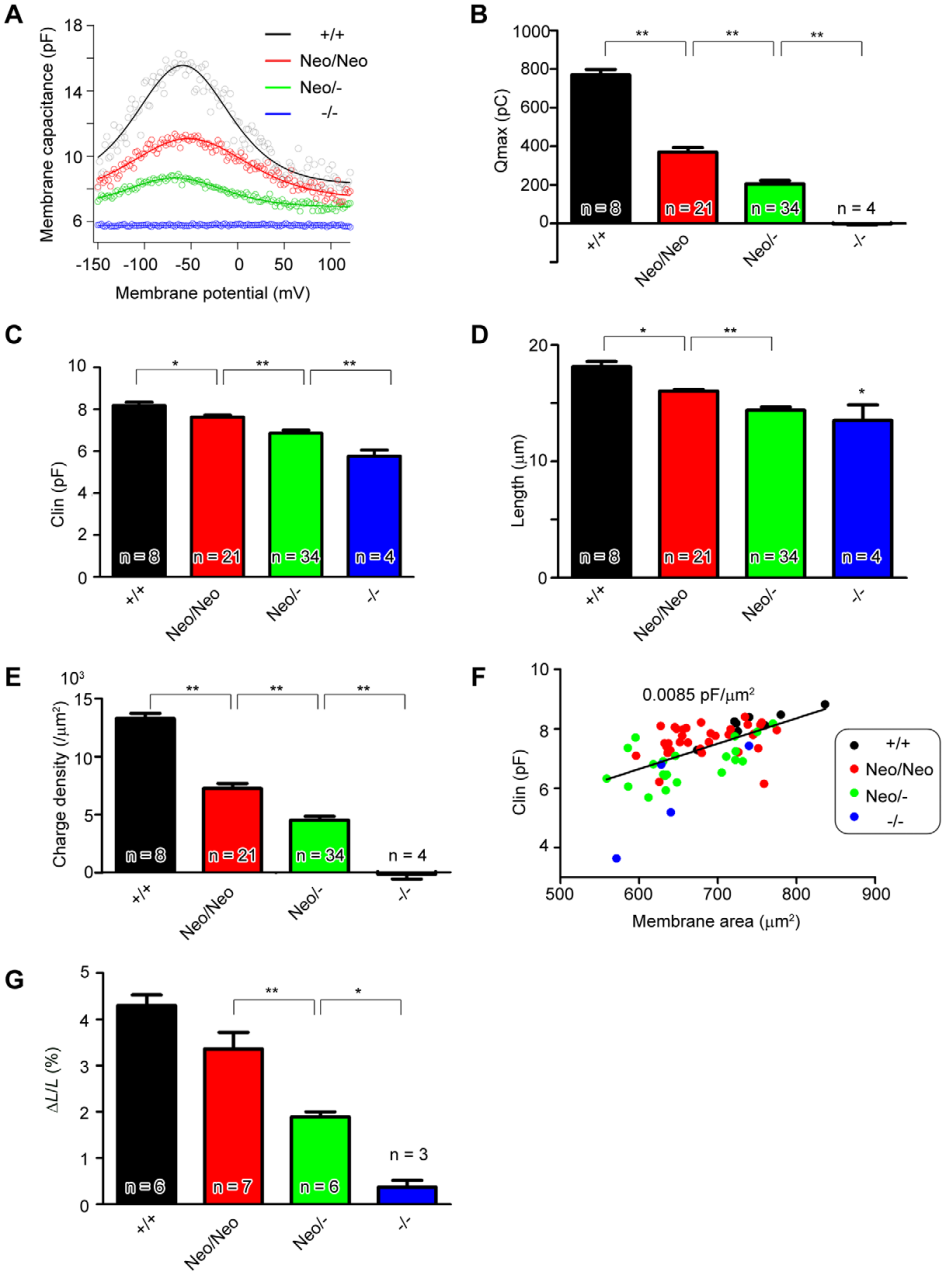
Membrane capacitance versus membrane potential and electromotility *in vitro*. (***A***) Voltage-dependent membrane capacitance of OHCs of the indicated genotypes, obtained at a voltage range of −150 mV to 120 mV. (***B***) Maximum charge transfer of OHCs of the indicated genotypes, expressed as absolute total charge transfer. Values in *Neo/Neo* and *Neo/-* OHCs are between those in wild-type and *prestin−/−* OHCs. (***C***) Linear membrane capacitance in the indicated OHC genotypes. Values in *Neo/Neo* and *Neo/-* OHCs fall between those in wild-type and *prestin−/−* OHCs. (***D***) Length of the OHC axial lateral wall in the indicated genotypes. Lengths were measured in the isolated cells in which voltage-dependent membrane capacitance (***A***) was obtained. (***E***) Estimated charge density in the indicted OHC genotypes. The area of the lateral membrane containing prestin was calculated as *A*_lat_ = π*DL* where *D* is the diameter and *L* is the length of the membrane. The charge density of OHCs was calculated by dividing each cell's *Q*_max_×1*C* (6.24×10^18^ elementary charges) by the total prestin-containing surface area. (***F***) Linear capacitance vs. the combined surface area of the lateral membrane area, the cuticular plate area, and the basal area in the indicated genotypes. The line represents the membrane capacitance of OHCs per unit of surface area (0.0085 pF/µm^2^). (***G***) Electromotile amplitude in the indicated genotypes. OHC length changes in response to voltage steps (−150 to 120 mV in 30-mV increments) were recorded in whole-cell, voltage-clamp mode. The absolute value of the amplitude was normalized to the OHC length at holding potential to allow comparison between genotypes. Bars show the mean (±SEM) maximum OHC motility expressed as percent length change. **: *P*<0.01, *: *P*<0.05 as determined by the Kruskal-Wallis test followed by Student's t test with a Holm correction.

To assess prestin's contribution to cochlear mechanical amplification, we measured the prestin amount in OHCs of *Neo/Neo* and *Neo/-* mice; while immunofluorescent measurements are semi-quantitative at best and not definitive (data not shown), we chose to quantify the prestin amount by using electrophysiological measurements of isolated OHCs that have been well established for such purposes. Specifically, we compared the levels of functional prestin by measuring NLC, charge density, electromotility, and hearing sensitivity in *Neo/Neo* and *Neo/-* mice at days P21–P24 with those of *+/+* and *−/−* mice. OHCs exhibited a bell-shaped NLC in response to the change in membrane potential. Because NLC has been associated with the voltage-dependent charge transfer within prestin [14], the expression level of functional prestin can be evaluated by measuring NLC. The capacitance data for each OHC was fitted using a derivative of a first-order Boltzmann function [14], [22]. Total elementary charge movements (*Q*_max_) in *Neo/Neo* and *Neo/-* OHCs were significantly lower than in *+/+* controls (Kruskal-Wallis test, *P*<0.001) but were between those in *+/+* and *−/−* OHCs at P21–24 (normalized to +/+, 47.9%±3.2% for *Neo/Neo*, 26.7%±2.1% for *Neo/-*, and −1.0%±3.7% for *−/−* OHCs; Fig. 3*A, B*). Linear capacitance (*C*_lin_) in *Neo/Neo* and *Neo/-* OHCs was significantly lower than that in *+/+* OHCs (Kruskal- Wallis, *P*<0.001) but not as low as that in *−/−* OHCs at P21–24 (93.3%±1.1% for *Neo/Neo*, 83.8%±1.8% for *Neo/-*, and 70.4%±10.4% for *−/−*; Fig. 3*C*).

To calculate charge density, the total surface area of the lateral membrane and the cell diameter of isolated OHCs used to obtain the NLC data were examined. The isolated OHC length was significantly lower in *−/−* mice than *+/+* controls (∼74.6%±7.4%; *P*<0.05, Kruskal-Wallis test; Fig. 3*D*). The length of the OHC axial lateral wall in *Neo/Neo* and *Neo/-* OHCs was significantly lower than that in wild-type controls (*P*<0.05, Kruskal-Wallis test) but was greater than that in *prestin−/−* OHCs (88.5%±0.7% and 79.4%±1.6%, respectively). The lengths of isolated and *in situ* OHCs shown previously in Figure 2 were significantly correlated (*r* ^2^ = 0.94, *P*<0.05). The OHC diameter did not differ significantly among genotypes (*P*>0.05, Kruskal-Wallis test; [17]). Mean (± SEM) diameters were 6.4±0.1 µm in wild-type, 6.3±0.1 µm in *Neo/Neo*, 6.3±0.1 µm in *Neo/-*, and 6.3±0.1 µm in *prestin−/−* cells. The mean (± SEM) surface areas of the lateral wall membrane were 362.8±11.6 µm^2^ in +/+, 315.0±3.6 µm^2^ in *Neo/Neo*, 285.1±7.2 µm^2^ in *Neo/-*, and 268.4±28.1 µm^2^ in *prestin−/−* OHCs (Fig. 3*E*). Therefore, the charge densities calculated by dividing each cell's *Q*_max_×1*C* (6.24×10^18^ elementary charges) by the total prestin-containing surface area were 13273.4±443.9/µm^2^ in +/+, 7248.4±431.5/µm^2^ in *Neo/Neo*, 4511.7±335.6/µm^2^ in *Neo/-*, and −139.6±421.1/µm^2^ in *prestin−/−* OHCs. The values for *Neo/Neo*, *Neo/-*, and *prestin−/−* OHCs differed significantly from those for +/+ controls (*P*<0.01, Kruskal-Wallis test). The charge densities of *Neo/Neo* and *Neo/-* OHCs were intermediate between that of +/+ controls and *prestin−/−* OHCs (54.6%±3.3% for *Neo/Neo*, 34.0%±2.5% for *Neo/-*, and 1.1%±3.2% for *prestin−/−* OHCs). These data demonstrate that the effective levels of prestin function in *Neo/Neo* and *Neo/-* OHCs are intermediate between those of *prestin−/−* and +/+ OHCs. Consistent with this, the prestin mRNA level of *Neo/-* mice at P60 was 27.4% (*n* = 2, range 5.9% to 49.9%) of that of +/+ mice.

The combined surface area of the lateral membrane (*A*_lat_), the cuticular plate area (*A*_cut_), and the basal area (*A*_bas_) in each genotype was also estimated and plotted against linear capacitance. The membrane linear capacitance per unit of area has been reported to be 0.008 pF/µm^2^ in developing OHCs of postnatal mice [23]. We calculated *A*_lat_ as π*DL*, *A*_cut_ as πD^2^/4, and *A*_bas_ as π*D* ^2^/2, where *D* is the diameter and *L* is the length of the membrane containing prestin. The membrane capacitance of OHC per unit surface area was 0.0085 pF/µm^2^ in all 4 genotypes of prestin (Fig. 3*F*), similar to that seen during development [23]. The voltage at peak capacitance (*V*_1/2_) did not differ significantly in *Neo/Neo* and *Neo/-* OHCs compared with wild-type controls (*P*>0.05, Kruskal-Wallis test; Table 1). Interestingly, the voltage dependence (α) in *Neo/Neo* OHCs was significantly different from that in wild type control (P<0.01, Kruskal-Wallis test; Table 1).

**Table 1:** Function parameters of OHCs from the 4 genotypes of mice.

Genotype	Q_max_ (fC)	Clin (pF)	α	V½ (mV)
*+/+* (n = 8)	769.85±28.44	8.19±0.16	36.01±0.56	−57.86±0.97
*Neo/Neo* (n = 21)	369.09±24.50**	7.64±0.09*	44.68±1.24 **	−51.44±3.24
*Neo/-* (n = 34)	205.72±16.36 **	6.86±0.15**	40.75±1.45	−62.54±3.01
*−/−* (n = 4)	−2.03±4.93**	5.77±0.29**	N.A.	N.A.

Note:

** : p<0.01,

* : p<0.05, compared to wild-type OHCs, as determined by the Kruskal-Wallis test followed by Student's t test with a Holm correction; NA: not applicable.

We further measured changes in OHC length in response to changes in membrane voltage (electromotility) in +/+, *Neo/Neo*, *Neo/-*, and *prestin−/−* OHCs (Fig. 3*G*). The maximum value of changes in OHC length was normalized to the length (ΔL/L) at holding potential of −70 mV. The amplitude of electromotility in *Neo/-* OHCs was significantly lower than that in +/+ controls (*P*<0.01, Kruskal-Wallis test) but was between that of +/+ and *prestin−/−* OHCs at P21–24 (78.2±8.3% for *Neo*/*Neo*, 44.0%±2.5% for *Neo/-*, 8.6%±3.4% for *prestin−/−* OHCs; Fig. 3*G*). Electromotile amplitude and charge density were significantly correlated (*r* ^2^ = 0.97, *P*<0.05) which is a characteristic of wild-type prestin. There was no significant difference in ΔL/L values between *Neo/Neo* and +/+ controls; however, linear regression analysis showed a gradient decrease in the amplitude in a prestin genotype–dependent manner (*r* ^2^ = 0.91, *P*<0.05). These results suggest that the electromotile amplitude in *Neo/Neo* OHCs is also at an intermediate level.

**Figure 4:**
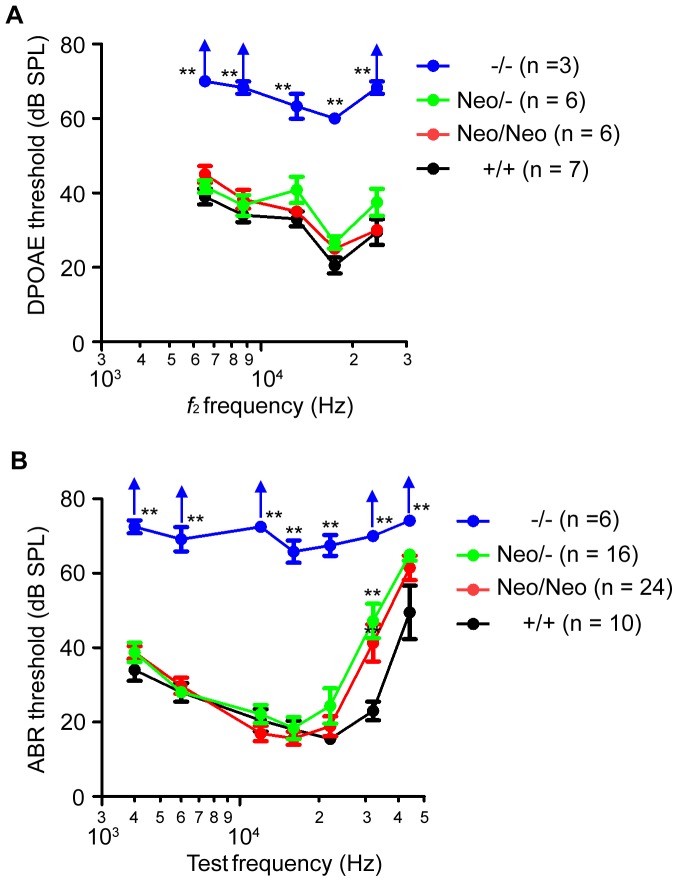
DPOAE (*A*) and ABR (*B*) thresholds of P21–24 mice of the indicated genotypes. Values are the mean ± SEM; **: *P*<0.01, *: *P*<0.05 by two-way ANOVA followed by Student's t test with a Bonferroni correction.

In order to examine OHC function *in vivo*, we measured the cubic 2*f_1_*−*f_2_* distortion product (where *f*_2_/*f*_1_ = 1.21 and *L*_2_ = *L*_1_−10 dB) of the otoacoustic emissions (DPOAE) in anesthetized P21–24 mice. In our setup, we could record accurately the DPOAE 2*f_1_*−*f_2_* responses at a frequency of *f_1_* range of 5454–18180 Hz and *f*_2_ range of 6599–21998 Hz (see methods). DPOAE thresholds in *−/−* mice were measurable and 30 to 40 dB higher than those in *+/+* controls at P21–24 (Fig. 4*A*). DPOAE thresholds in *prestin−/−* mice at 6–8 weeks have been reported to be elevated by 45 to 55 dB compared to *+/+* controls [2], [24]. In *prestin−/−* mice, spotty hair-cell loss has been reported to start between P21 and P28, and almost all OHCs are lost at P42 [2], [3]. The differences are likely related to age. Alternatively, this could be because of systematic differences such as noise floors and different calibration method between laboratories. Remarkably, we found no statistically significant difference in the DPOAE thresholds in *Neo/Neo* and *Neo/-* mice versus *+/+* controls at any of the frequencies analyzed (Fig. 4*A*). These results indicate that *in vivo* OHC function in *Neo/Neo* and *Neo/-* mice is comparable to that in *+/+* controls at least in low-to-middle frequencies (4–22 kHz).

In order to assess *in vivo* amplification gain for *Neo/Neo* and *Neo/-* mice, we further examined ABR thresholds. To avoid age-related hearing loss at high frequencies, we measured ABR thresholds at as early as P21–P24. During the course of our studies, we adopted two different conditions: open and closed fields (see Methods), both of which yielded similar ABR thresholds of *+/+* mice among various crosses in several generations. In Figure 4B, all data points of each genotype were therefore combined. ABR thresholds of *Neo/Neo* and *Neo/-* mice did not differ significantly from those of *+/+* mice at frequencies 4–22 and 44 kHz tested (except at 32 kHz, ANOVA, P<0.05, Fig. 4B). The hearing sensitivities were variable at 32–44 kHz in *Neo/Neo* and *Neo/-* mice (ANOVA, P<0.05; data not shown). This could be due to genetic variation and/or genetic drift or aging, consistent with previous reports [2], [8], [24]. Together, *Neo/Neo* and *Neo/-* mice exhibited wild-type-like hearing sensitivities at least in low-to-middle frequencies at 4–22 kHz, consistent with our DPOAE measurements (Fig. 4A).

## Discussion

**Figure 5:**
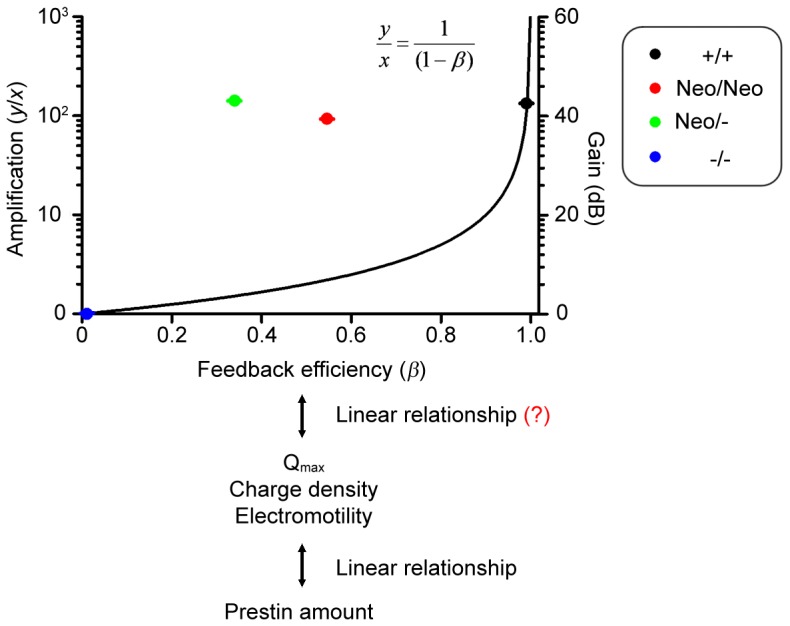
The relationship between prestin activity/feedback efficiency and amplification gain. The relationship between amplification gain and feedback efficiency proposed by Patuzzi et al. [7] is plotted as a black curve. Y-axes represent the input/output (y/x) ratio (left) and the amplification gain [dB SPL = 20log(y/x), right], respectively. The amplification gain (dB SPL) for each genotype is the ABR threshold difference between prestin *−/−* mice and wild-type, *Neo/Neo*, or *Neo/-* mice. Mean values derived from our data are color-coded according to genotype. In this study, averaged ABR threshold changes at 16 kHz were used to derive the amplification gain, although similar results were obtained at frequencies of 4–12 and 22 kHz. We assumed that the prestin amount was linearly correlated with *Q*_max_, charge density or electromotility, which is further linearly correlated with feedback efficiency. Therefore, the normalized prestin activity in *Neo/Neo* and *Neo/-* OHCs by wild-type control (100% prestin activity) is expressed as the feedback efficiency (β). Values are the mean ± SEM.

In existing models of cochlear amplification [7], the contribution of OHCs to BM vibration is seen as a simple feedback system in which the output (*y*) is related to the input (*x*) according to the equation *y* = *x*(1−β)^−1^, where β is the feedback efficiency (Fig. 5). Assuming a linear correlation between the feedback efficiency and the activity of prestin (*Q*_max_, charge density or electromotility), a nonlinear correlation between charge density/electromotility and amplification gain can be predicted (Fig. 5).

Normal mammalian cochleae have 100% prestin activity, amplify sounds by 45–65 dB, and feedback efficiency is expected to be approximately 100% [99.40% to 99.43% as calculated by the equation *y* = *x*(1−β)^−1^]. Consistent with this model, the complete absence of prestin in *prestin*−/− mice is likely to break the feedback loop (i.e., 0% efficiency), thus preventing amplification [2], [3], [19], [24]. However, this prediction is in contrast to the originally reported 74% level of functional prestin (as *Q*_max_), 83% charge density, 56% electromotility, and approximately 6 dB loss in *prestin+/−* mice at 5–8 weeks of age [2], [17]. Another study demonstrated that the prestin expression is upregulated in *prestin+/−* mice, thus providing undiminished charge density and electromotility to support the wild-type–like amplification gain [8]. Possibly the differences between these results are due to genetic drift in subsequent generations of *prestin+/−* mice [2], [8], [17].

Several previous studies have indirectly explored this relationship *in vivo*. Systemic injection of furosemide, resulting in a 50% (6 dB) reduction of receptor currents, causes the hearing threshold to increase by 25–30 dB [25]; intravenous injection of quinine, which may affect OHCs to a certain extent, also reduces sensitivity by up to 15 dB [26]. Perilymphatic perfusion of the cochlea with sodium salicylate gradually reduces sensitivity by up to 45 dB [27]. Because sodium salicylate reversibly blocks OHC electromotility, the kinetics of this reduction in sensitivity during perfusion may provide some insight into the correlation between OHC electromotility and cochlear amplification. More recently, prestin function was found to be diminished by cochlear perfusion with a weak Cl^−^ solution, which led to loss of gain [28]. However, these drugs or ion replacement treatments have a wide range of effects in the cochlea, making it difficult to directly quantify the effect of OHC electromotility in the feedback mechanism *in vivo*.

In another study, prestin chimeric mice with various ratios of OHCs containing wild-type and knockout prestin displayed a nearly linear relationship between the percentage of OHCs expressing wild-type prestin and cochlear sensitivity gain [20]. However, wild-type OHCs were mixed with various numbers of *prestin*−/− OHCs in a mosaic manner in these chimeric mice and the structure of their organ of Corti differed from that of wild-type mice, thus complicating the interpretation of the findings; in fact, the data only minimally deviated from the predicted Patuzzi's model.

In our study, we created knock-in mice that expressed wild-type-like prestin (i.e., wild-type-like voltage at peak capacitance (*V*_1/2_) for *Neo/Neo* and *Neo/-*, and wild-type-like voltage dependence (α) for *Neo/-*, Table 1), but at reduced levels (*Q*_max_, 47.9% in *Neo/Neo*, 26.7% in *Neo/-*), and that showed reduced charge density (54.6% in *Neo/Neo*, 34.0% in *Neo/-*) and electromotility (78.2% in *Neo/Neo*, 44.0% in *Neo/-*). The gross structure of OHCs and the organ of Corti remained intact despite reduction in OHC length at P21–P24 [2], [24]. We conducted at least 2 independent measurements of several critical parameters: cell length in isolated OHCs and whole-mount preparations; charge density through linear capacitance-derived and direct measurements of cell membrane area; and hearing sensitivity by both ABR and DPOAE. Moreover, all measurements (NLC, electromotility, ABR, DPOAE) in our study in *+/+* and *prestin−/−* mice were consistent with those of previous studies [2], [3], [8], [17], [24]. It is noted that charge density and electromotility measurements from the same mouse strain, albeit similar overall, were not entirely consistent under our *in vitro* conditions; such disparity between charge density and electromotility in similar OHCs has been reported previously [2], [17].

It is noted that charge density is similar, but not identical to prestin density. To derive the prestin density from the prestin activity related to measured NLC, the valence (z) was calculated by the equation α = ze/KT, where K is Boltzmann's constant, T is absolute temperature, z is valence of charge movement and e is electron charge. The valences of charge movement by prestin (z) were 0.71±0.01 in *+/+*, 0.58±0.02 in *Neo/Neo*, and 0.64±0.02 in *Neo/-*. Consistent with α value, the valence in *Neo/Neo* OHCs was significantly different from that in +/+ control (p<0.01, Kruskal-Wallis test). The prestin density was further corrected by dividing α with z and the densities were significantly different from +/+ control (70.4±6.5% in *Neo/Neo* and 39.6±4.1% in *Neo/-* OHCs after normalized to *+/+*; p<0.05, Kruskal-Wallis test). In support, immunogold measurements of prestin density in the lateral wall of OHCs were correlated with measurements of charge density in isolated OHCs [29]. Moreover, there are little differences in prestin density between low and high frequency regions of cochleae [30].

*Q*_max_ is considered to be an accurate measurement of total amount of functional prestin and charge density an accurate indicator of prestin activity in OHCs [8]. Interestingly, we observed a uniform gradient of OHC cell length along the cochleae in each genotype (Fig. 2F), supporting that changes in charge density affect OHCs uniformly along the cochlear length and that prestin activity is uniform along cochlear length in each mutant mouse. Although the *Q*_max_ and OHC cell length vary along the cochlear length in each mutant mouse [29], [30], electromotility measured in isolated OHCs may exert different forces between apical and basal turns in the intact cochlea because of their different morphology and geometric orientations relative to the BM. Therefore, among the four parameters we measured (*Q*_max_, charge density, prestin density, and electromotility), it is reasonable to use charge density as the best indicator of prestin activity and thus feedback efficiency.

Therefore we expect 34.0% and 54.6% of normal prestin activity (as charge density) to result in a 38.1–41.4 dB loss of amplification if we assume that wild-type cochleae achieved 45 dB of amplification (Fig. 5; N. Cooper, personal communication; [7], [31], [32]). Surprisingly, we found that the hearing sensitivity of mice (*Neo/Neo* and *Neo/-*) was comparable to that of *+/+* controls at least in low-to-middle frequency cochlear regions (4–22 kHz). It is noted that all our *in vitro* OHC measurements (*Q*_max_, charge density and electromotility) were obtained in similar low-to-middle frequency regions. These striking results led us to conclude that: (1) *in vivo* neuronal activity generated by IHCs (based on ABR measurements) and OHC activity (based on DPOAE measurements) are not linearly correlated with prestin amount or *in vitro* activity (NLC, charge density, and electromotility); and (2) as low as 26.7% of *Q*_max_, 34.0% charge density, and 44.0% electromotility compared to the wild-type levels are sufficient for wild-type–like hearing sensitivity at least at 4–22 kHz.

The simplest explanation for our findings is that the assumption that there is a linear correlation between the feedback efficiency and the prestin activity (charge density) is inaccurate. Many factors could disaccord the linearity between prestin charge density and feedback efficiency. For example, the charge density may not be uniform along the lateral wall of OHCs [30] although there is a discrepancy between uniform and non-uniform distribution of prestin along lateral wall of OHCs [29]. Therefore, not all prestin charge movements in the same OHC contribute equally to feedback efficiency. In addition, prestin can exist as monomers or oligomers [5], [17], [33] and can be subject to protein modifications such as phosphorylation, thus contributing to the feedback efficiency differently in different cellular or sub-cellular environment. Moreover, prestin activity has traditionally been defined by *in vitro* measurements of isolated OHCs, a hallmark of OHCs that remains to be demonstrated *in vivo*.

A second explanation for our striking results is that other components of OHCs may compensate for the reduction of prestin activity to provide the appropriate feedback for cochlear amplification [1]. Indeed, exposure of methyl-β-cyclodextrin, which depletes membrane cholesterol, in temporal bone preparation increased vibration of organ of Corti in amplitude [34]. The depletion of the cholesterol induced depolarizing shift of V_1/2_ [35]. However, no differences were observed in V_1/2_ from *Neo/Neo* and *Neo/-* OHCs compared with wild-type controls (Table 1). It would be interesting to directly measure axial stiffness of OHCs in all prestin mouse models which may hold the key to resolve the apparent discord between our findings and the Patuzzi model, given that axial stiffness was reduced in prestin *−/−* mice [5]. It has been hypothesized that prestin associates with actin cytoskeleton [36]. Therefore, reduced prestin amount could cause changes in mechanical properties of OHCs through altering cytoskeletal stiffness or indirectly efferent fiber activity. Recent power efficiency analyses reveal length dependence when the piezoelectric-like conversion of electrical to mechanical energy is considered [37], [38]. Therefore, a shortening of OHCs could be a compensatory mechanism shifting the motor mechanism towards a higher frequency. It remains possible that such a strategy may only work for the low to middle frequencies but not for the higher frequencies. Regardless of interpretations, our results provide valuable data for modeling cochlear mechanics.

At 32 kHz we observed significant increase of ABR thresholds in *Neo/Neo* and *Neo/-* mice compared to *+/+* littermates at as early as P21–P24 in a large number of mice in several successive generations (Fig. 4B). We also observed significant elevations in ABR thresholds at 44 kHz in all genotypes. Because of the mixed strain backgrounds between C57BL/6 and 129SvEv in our models, such strain background effects on age-dependent high-frequency hearing loss may play determinant roles in our ABR threshold results in high frequency regions. Therefore, at present time we cannot determine whether or not our prestin hypomorphic mouse models indeed exhibit cochlear gain loss at high frequencies (>22 kHz). Future measurements in other strain backgrounds that are less prone to age-related high frequency hearing loss (i.e., CBA/CaJ) may provide further insights into mechanisms of cochlear amplification in our models.
